# Lactate as a metabolite from probiotic *Lactobacilli* mitigates ethanol-induced gastric mucosal injury: an in vivo study

**DOI:** 10.1186/s12906-020-03198-7

**Published:** 2021-01-11

**Authors:** Yingpeng Huang, Jiali Zhang, Renjie Dong, Xiawei Ji, Yusha Jiang, Jianke Cen, Zhihuai Bai, Kairui Hong, Huihui Li, Jiajing Chen, Jinhui Zhou, Fanyu Qian, Fangyan Wang, Yue Qu, Yan Zhou

**Affiliations:** 1 The Second Affiliated Hospital and Yuying Children’s Hospital of Wenzhou Medical University, Wenzhou, China; 2 Department of Pathophysiology, School of Basic Medical Science, Wenzhou Medical University, Wenzhou, China; 3 Wenzhou Key Laboratory of Sanitary Microbiology, Key Laboratory of Laboratory Medicine, Ministry of Education, China, School of Laboratory Medicine and Life Sciences, Wenzhou Medical University, Wenzhou, 325035 China; 4 Department of Microbiology, Biomedicine Discovery Institute, Monash University, Clayton, 3800 Australia

**Keywords:** Lactate, Ethanol, Gastric mucosal injury, Inflammation, Apoptosis, Tight junction proteins

## Abstract

**Background:**

Pre-administration of probiotic *Lactobacilli* attenuates ethanol-induced gastric mucosal injury (GMI). The underpinning mechanisms remain to be elucidated. We speculated that lactate, the main metabolite of *Lactobacillus* that can be safely used as a common food additive, mediated the gastroprotective effect. This study aimed to gain experimental evidence to support our hypothesis and to shed lights on its underlying mechanisms.

**Methods:**

Lactate was orally administrated to mice at different doses 30 min prior to the induction of GMI. Gastric tissue samples were collected and underwent histopathological and immunohistochemical assessments, enzyme-linked immunosorbent assay, quantitative polymerase chain reaction (qPCR) and western blot analyses.

**Results:**

Pretreatment with lactate at 1–3 g/kg significantly curtailed the severity of ethanol-induced GMI, as shown by morphological and histopathological examinations of gastric tissue samples. Significantly lower level of cytokines indicative of local inflammation were found in mice receiving lactate treatment prior to ethanol administration. Western-blot, immunohistochemical analysis and qPCR suggested that gastroprotective properties of lactate were mediated by its modulatory effects on the expression of the apoptosis regulator gene *Bax*, the apoptotic executive protein gene *Casp3*, and genes critical for gastric mucosal integrity, including those encoding tight junction proteins Occludin, Claudin-1, Claudin-5, and that for lactate receptor GPR81.

**Conclusion:**

Lactate mitigates ethanol-induced GMI by curtailing local gastric inflammatory response, down-regulating the expression of the apoptosis regulator and executor genes *Bax* and *Casp3*, and up-regulating the expression of genes encoding tight junction proteins Occludin, Claudin-1, and Claudin-5 and the lactate receptor GPR81.

**Supplementary Information:**

The online version contains supplementary material available at 10.1186/s12906-020-03198-7.

## Background

Gastric mucosal injury (GMI) is a common precancerous condition related to the high incidence of gastric cancer in many Asian countries, including China [1–4]. Current treatment strategy for this troublesome medical condition focuses on preventing gastric mucosa from acid erosion, rather than actively repairing the damaged mucosa [2, 5].

It is known that gastric mucosal injury is associated with elevated local inflammation in the gastric mucosa, with inflammatory cytokines such as IL-1β, TNF-α and IL-6 all being overexpressed [6, 7]. Apoptosis is a key mechanism driving the pathogenesis of gastric mucosal injury [8]. Bcl-2-associated X (Bax) is a cytosolic protein that permeabilizes mitochondrial outer membrane and has been considered as one of the core regulators of the intrinsic apoptosis pathway [7, 9]. Caspase 3 is the key executor protein for the caspases cascade reaction of apoptosis, interacting with caspase-8 and caspase-9 [9, 10]. Numerous in vivo studies have reported that many genera of probiotic *Lactobacilli* had protective effects on the gastric mucosa; the underlying mechanisms, however, remain to be fully elucidated [11–16]. Lactate is a major metabolite of *Lactobacilli* that has been widely used as a food additive for human [17, 18]. Using different disease models, Hoque et al. (2014) and Ranganathan et al. (2018) both found that lactate was able to attenuate intestinal inflammation via endothelial lactate-receptor GPR81 signaling pathway [19, 20].

Long-term or excessive drinking alcohol is an important cause of GMI [21, 22]. Orally administration of absolute ethanol into small animals such as mice has been proven to be able to cause gastric symptoms closely mimicking that of humans [23, 24]. The mouse model of ethanol-induced GMI has been extensively used for anti-gastric ulcer drug screening and other mechanistic studies [24, 25]. By adopting this well-established mouse model, the aims of this study were to assess the gastroprotective effect of lactate and to investigate its underlying mechanisms.

## Methods

### Reagents, chemicals and kits

L-lactate sodium was purchased from Sigma-Aldrich Co. (St. Louis, USA). Commercial kits for bicinchoninic acid (BCA) protein assay and Trizol were from Beyotime Institute of Biotechnology (Nantong, China). Enzyme-linked immunosorbent assay (ELISA) kits for IL-1β, TNF-α and IL-6 were from Westang Bio-tech Co., Ltd. (Shanghai, China). PrimeScript RT reagent Kit for reverse transcription and SYBR Green were both from TaKaRa Co., Ltd. (Kusatsu, Japan). The primary antibody against Bax was purchased from Biosynthesis Biotechnology Co. Ltd. (Nanijing, China) and the primary antibody for Caspase 3 was from Proteintech Group, Inc. (Wuhan, China), and the primary antibody for GPR81 was form Affinity Bioaciences (Beijing, China).

### Animal groups and lactate pretreatment

Male Institute of Cancer Research (ICR) mice (10 weeks, 25–30 g) were purchased from Experimental Animal Center of Wenzhou Medical University (Wenzhou, China). This study was approved by the Animal Care and Ethics Committee of Wenzhou Medical University, China (Ethics approval number: wydy2012–0109). All experiments were performed in accordance with the guidelines and regulations of the Committee for the use and care of animals.

Fifty mice were randomly divided into 5 groups (10 mice per group), including a baseline control group without any treatment, a GMI–induced disease control group and three treatment groups pre-treated with low (1 g/kg), intermediate (2 g/kg) and high (3 g/kg) dosages of lactate via an orogastric tube 30 min prior to GMI modeling. Equal volume of NaHCO3 solution pre-adjusted to the same pH as lactate solutions were orally given to animals in the disease group.

### Ethanol-induced GMI model

Mice were fasted for 24 h with only water given ad libitum before GMI establishment. Absolute ethanol at 0.1 mL/g was orally given to mice in the disease group and treatment groups [5]. Animals were humanely euthanized using sodium pentobarbital (150 mg/kg, intraperitoneal) 1 h after ethanol administration and gastric tissue samples were collected.

### Visual examination of GMI

GMI of the gastric inner surface was visually examined and imaged with a digital camera (D7000, Nikon, Tokyo, Japan). Percentages of hemorrhagic lesion area to the total area of the studied gastric mucosa were calculated using acquired images and the Image-Pro Plus (IPP) 6.0 software.

### Histopathological analysis of GMI

For histopathological examination, gastric tissues were fixed in 4% paraformaldehyde overnight. Selected tissue blocks were processed using a routine overnight cycle in a tissue processor. The tissue blocks were then embedded in wax, serially-sliced into 5 μm sections. The transverse sections were stained with Hematoxylin–Eosin (HE) for tissue damage, visualized and imaged under a light microscope (Nikon ECLPSE 80i, Tokyo, Japan).

### Detection of cytokines in gastric tissues

Concentrations of three representative inflammatory effectors in the gastric tissue homogenate, including innate cytokines IL-1β, TNF-α and IL-6, were examined using commercially available enzyme-linked immunosorbent assay (ELISA) kits per instructions from the manufacturer [5]. Enzyme immunoassay (EIA) plates were incubated with dilutions of gastric tissue homogenate and serially diluted protein standards for 2 h. After washing, the plates were treated with biotinylated polyclonal goat anti-mouse IL-1β, TNF-α and IL-6 respectively for 2 h, followed by incubation with streptavidin horseradish peroxidase (HRP) for 20 min. A tetramethylbenzidine-H_2_O_2_ substrate solution was added to the plates, and the reactions were measured with a microplate reader at 450 nm.

### Immunohistochemistry

Gastric tissue blocks from different groups were fixed in 10% formalin and embedded in paraffin. Sections of 5 μm in thickness were prepared for immunohistochemical (IHC) analysis. Rabbit polyclonal antibodies against Bax and GPR81 were used. Antigen retrieval was achieved by high pressure in a citrate buffer (pH 6.0). The bound antibody was developed with diaminobenzidine (DAB) using a Dako REAL Envision staining kit (K5007) according to the manufacturer’s instruction. Stained sections were examined under a light microscope by two independent observers.

### BCA assay, SDS-PAGE and Western blot analysis

Concentrations of the total proteins extracted from gastric cancer samples were determined by BCA assay per instructions from the manufacturer. Protein samples (20 μg/lane) were separated using SDS-PAGE electrophoresis and then electrophoretically transferred to polyvinylidene fluoride (PVDF) membranes (Millipore, Billerica, USA). After blocking with 5% skim milk for 2 h at the room temperature, membranes were incubated with primary antibodies at 4 °C overnight. Antibodies against Bax, β-Actin, Caspase-3 and GAPDH were diluted with Primary Antibody Dilution Buffer (Beyotime Institute of Biotechnology) (1:1000 dilution). The membranes were then washed with PBST buffer five times (5 min each) and incubated with secondary antibodies for 2 h at the room temperature. The bands were detected using enhanced chemiluminescence and visualized with a ChemiDoc MP Imaging System (BioRad, Hercules, USA).

### RNA extraction and quantitative PCR

Total RNA was extracted from gastric tissues using TRIzol Reagent following the manufacturer’s instructions (Roche, Basel, Switzerland). For qPCR, reverse transcription was carried out with the PrimeScript RT reagent Kit. qPCR reactions were prepared using SYBR Green (TaKaRa) on a Prism 7500 Sequence Detector. The expression levels of mRNAs of IL-1β, TNF-α, IL-6, Occludin (OCLN), Claudin (CLDN)-1, CLDN-5, CLDN-18, ZO-1, tight junction protein 2 (TJP2), and GPR81 were all normalized to the level of glyceraldehyde 3-phosphate dehydrogenase GAPDH encoding gene TDH3. Sequences for qPCR primers for all targeted genes used in this study were listed in Table 1.

**Table 1:** Specific primers used for amplification of targeted genes

Gene name	Forward primer	Reverse primer
IL-1β	5′- GGAGAACCAAGCAACGACAA AATA −3’	5′- TGGGGAACTCTGCAGACTCAAAC − 3′
TNF-α	5′- TGGCCCAGACCCTCACACTCAG − 3′	5′- ACCCATCGGCTGGCACCACT − 3′
IL-6	5′- TGCCTTCTTGGGACTGAT − 3′	5′- TTGCCATTGCACAACTCTTT − 3’
Occludin	5′- TGAAAGTCCACCTCCTTACAGA − 3’	5′- CCGGATAAAAAGAGTACGCTGG − 3’
Clauldin-1	5′- TGTTCTTTTTAACCCCATGTGTCTT − 3’	5′- CACAGCTCAGAAACAGGAGGACT − 3’
Clauldin-5	5′- GAACAGACTACAGGCACTT − 3’	5′- TGGACATTAAGGCAGCAT − 3’
Clauldin-18	5′- TGTCTTACCATGCCTCTG − 3’	5′- ACTGTTCATCGTCTTCTGT − 3’
ZO-1	5′- GAGCGGGCTACCTTACTGAAC-3’	5′-GTCATCTCTTTCCGAGGCATTAG-3’
TJP-2	5′- CCGTGAGGATCGGGAATGAG − 3’	5′-GCTCTTGCGGAGGTTCTTCT-3’
GAPDH	5′- AGGTCGGTGTGAACGGATTTG − 3’	5′- GGGGTCGTTGATGGCAACA − 3’
Bax	5′-ACCAAGAAGCTGAGCGAGTG-3’	5′-CCCAGTTGAAGTTGCCATCA-3’
Caspase 3	5′-ATGGGAGCAAGTCAGTGGAC-3’	5′-GTCCACATCCGTACCAGAGC-3’
GPR81	5′-ATCCTGGTCTTCGTGCTTGG-3’	5′-CTGTCCGAAGGGGTAAGCAG-3’

### Statistical analysis

All experimental data were expressed as mean ± standard deviation (SD). Bonferroni test was used to compare differences between individual groups, with a *P*-value less than 0.05 considered statistically significant.

## Results

### Lactate reduced the severity of ethanol-induced GMI in mice

**Fig. 1:**
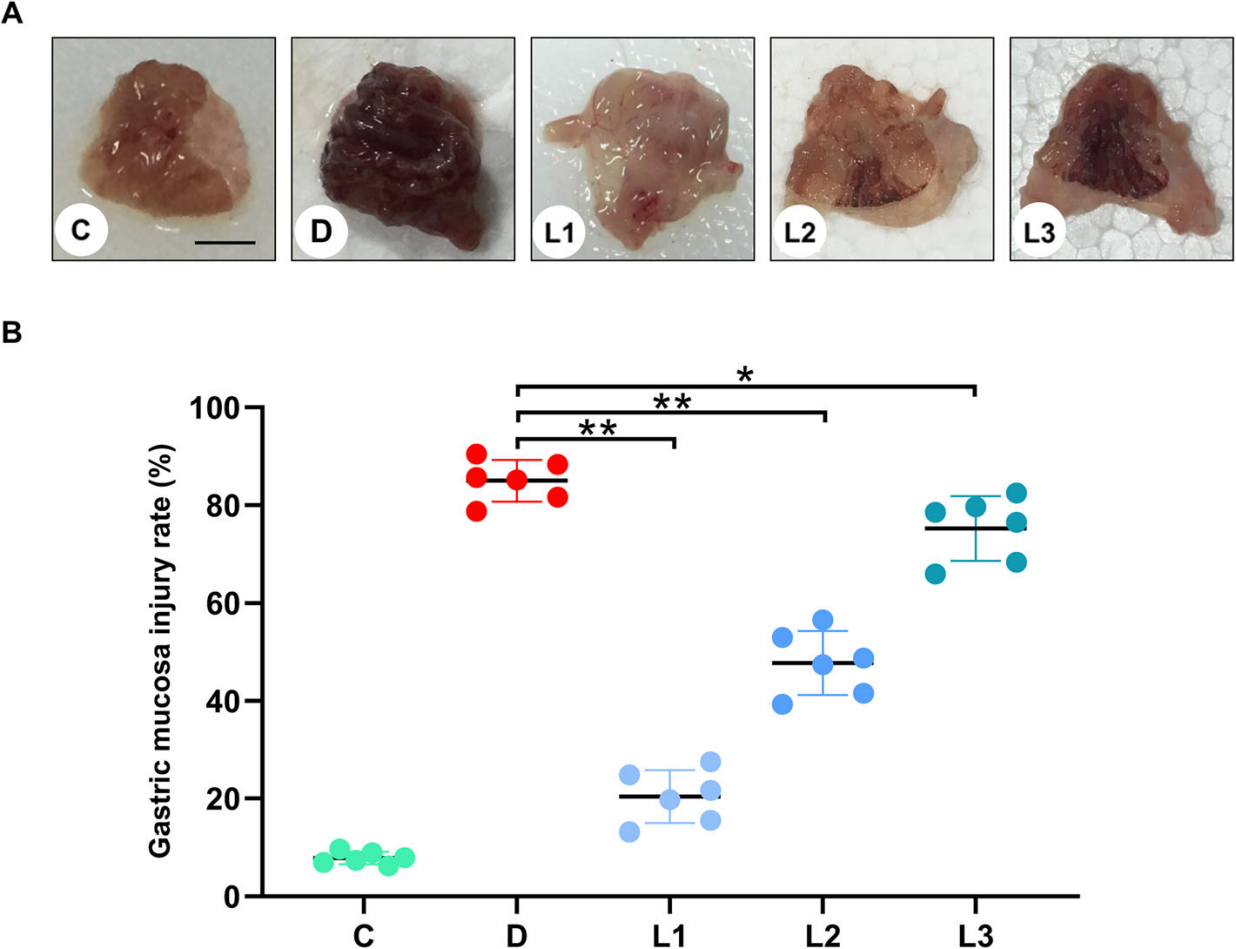
Effects of lactate on gross morphology and relative ulcer size of gastric mucosa in ethanol-challenged mice. **a** Gross morphological changes of gastric mucosa of different groups of mice (scale bar = 5 mm). **b** Relative ulcer sizes in gastric mucosa of different groups of mice. Relative ulcer sizes were expressed as ratios of the area of hemorrhagic lesion to the total area of the studied gastric mucosa. C: Control group; D: Disease group; L1-L3 represent the groups treated with lactate by the dose of 3 g/kg, 2 g/kg and 1 g/kg, respectively. The data are expressed as the means ± SD (*n* = 10). *: *p* < 0.05, **: *p* < 0.01

Extensive hemorrhagic injuries of the gastric mucosa were observed in mice that have been orally given absolute ethanol at 0.1 mL/ g (Fig. 1a). No evident lesion was found in the gastric mucosa from the control group (Fig. 1a). Pretreating mice with lactate prior to GMI induction with ethanol significantly reduced the severity of GMI in a dose-dependent manner, supported by both qualitative (Fig. 1a) and quantitative (Fig. 1b) image analysis.

**Fig. 2:**
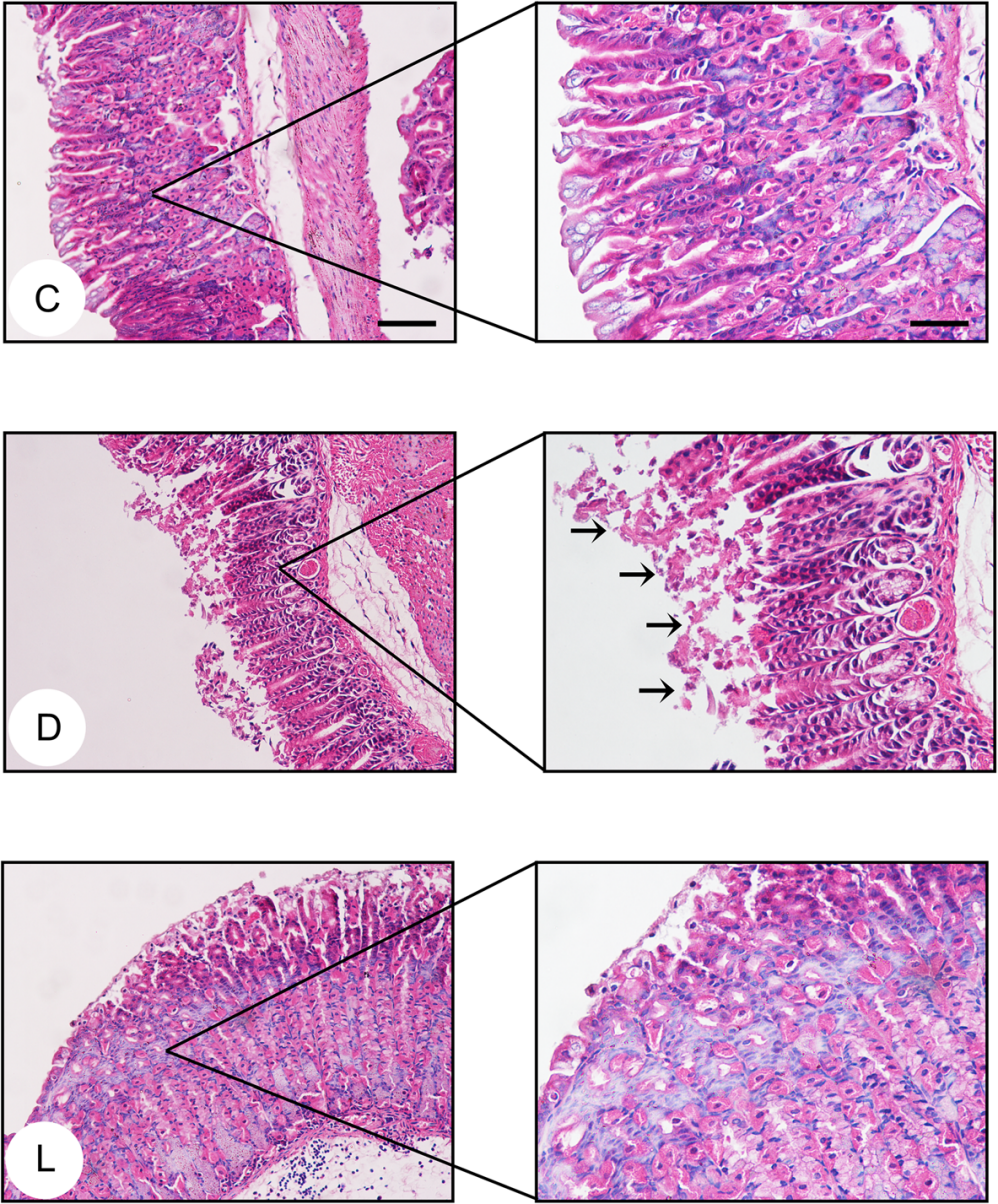
Effects of lactate on the histopathological changes of gastric mucosa in ethanol-challenged mice. Gastric tissue sections (*n* = 4 per group) were stained with hematoxylin and eosin (HE) followed by examination with a microscope (× 20, scale bar = 100 μm; × 40, scale bar = 50 μm). Black arrows showed the denudation of surface epithelium by ethanol challenge alone. C: Control group; D: Disease group; L: Group treated with lactate at a dose of 3 g/ kg

Given that high-dose of lactate showed the highest gastroprotective effect, it was decided to use gastric tissues from animals that received lactate at 3 g/ kg for detailed histopathological examination. In agreement with gross morphological changes, histopathological analysis of gastric tissue samples from animals pretreated with lactate showed less necrotic damage of the mucosa, less local mucosal detachment and less leukocyte infiltration, suggesting attenuated gastric mucosal damage (Fig. 2).

### Lactate mitigated excessive inflammation encountered in GMI

**Fig. 3:**
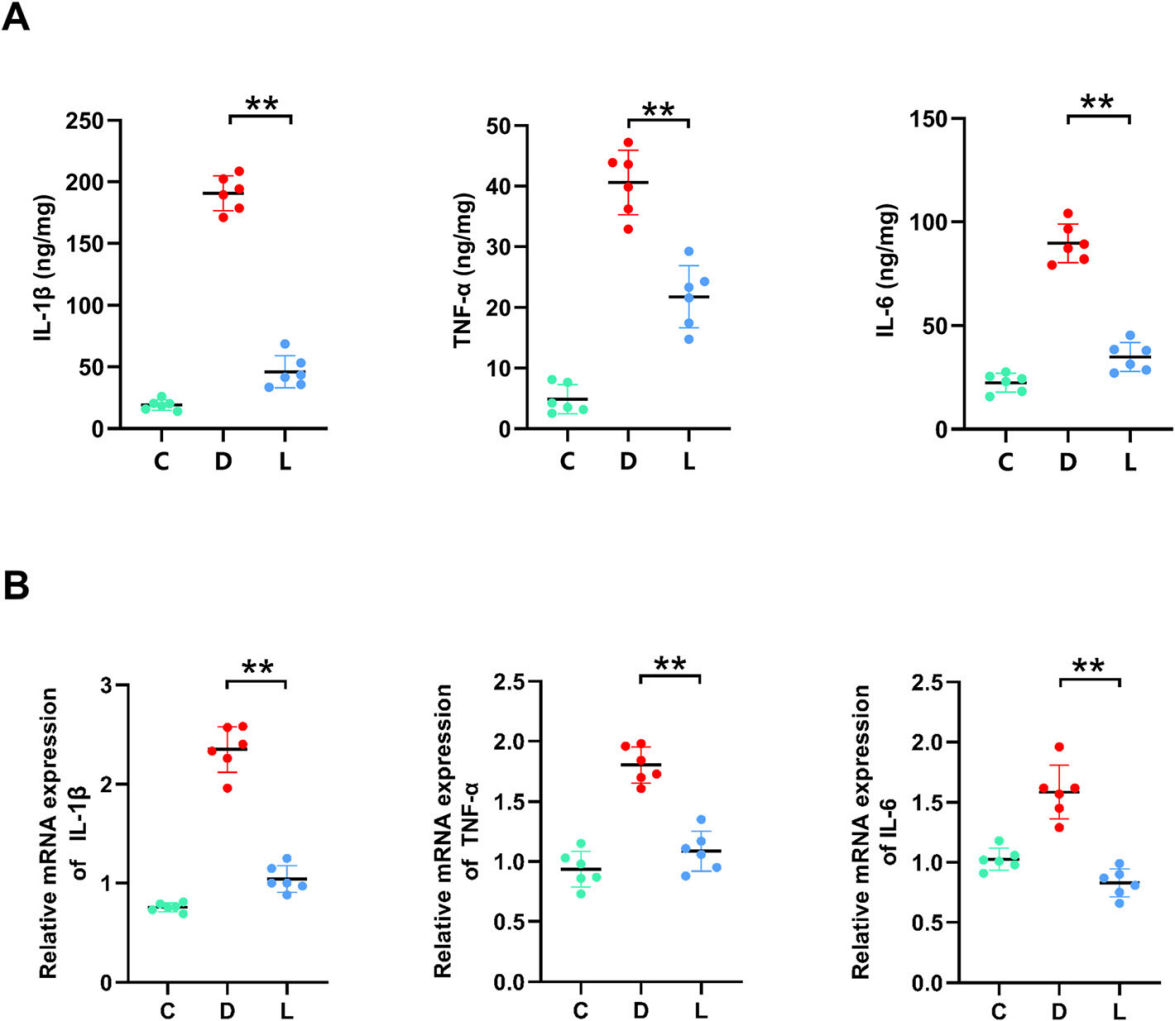
Effect of lactate on the inflammation of gastric tissues in ethanol-challenged mice. **a** ELISA for IL-1β, TNF-α and IL-6 in the gastric tissues. Gastric tissue homogenate was prepared for cytokines determination by ELISA. **b** qPCR for the expression of genes encoding IL-1β, TNF-α and IL-6 in the gastric tissues. C: Control group; D: Disease group; L: Group treated with lactate at 3 g/kg. The data were expressed as the means ± SD (*n* = 6). ** *p* < 0.01

Anti-inflammatory effect of lactate was determined by examining the level of pro-inflammatory cytokines IL-1β, TNF-α and IL-6 in homogenized gastric tissues. Exposure to ethanol triggered overproduction of all three pro-inflammatory cytokines in gastric tissues relative to untreated controls (Fig. 3a). Pretreating animals with lactate prior to ethanol exposure significantly mitigated the over-production of all three pro-inflammatory effectors (vs. disease group, *p* < 0.01, Fig. 3a). This was further supported by qPCR results, which showed over-expression of genes encoding IL-1β, TNF-α and IL-6 in the gastric tissues induced by ethanol exposure and down-regulation of the overexpression of these genes by lactate pretreatment (Fig. 3b).

### Lactate treatment led to less production of Bax and Caspase 3 in the gastric mucosa of ethanol-challenged mice

**Fig. 4:**
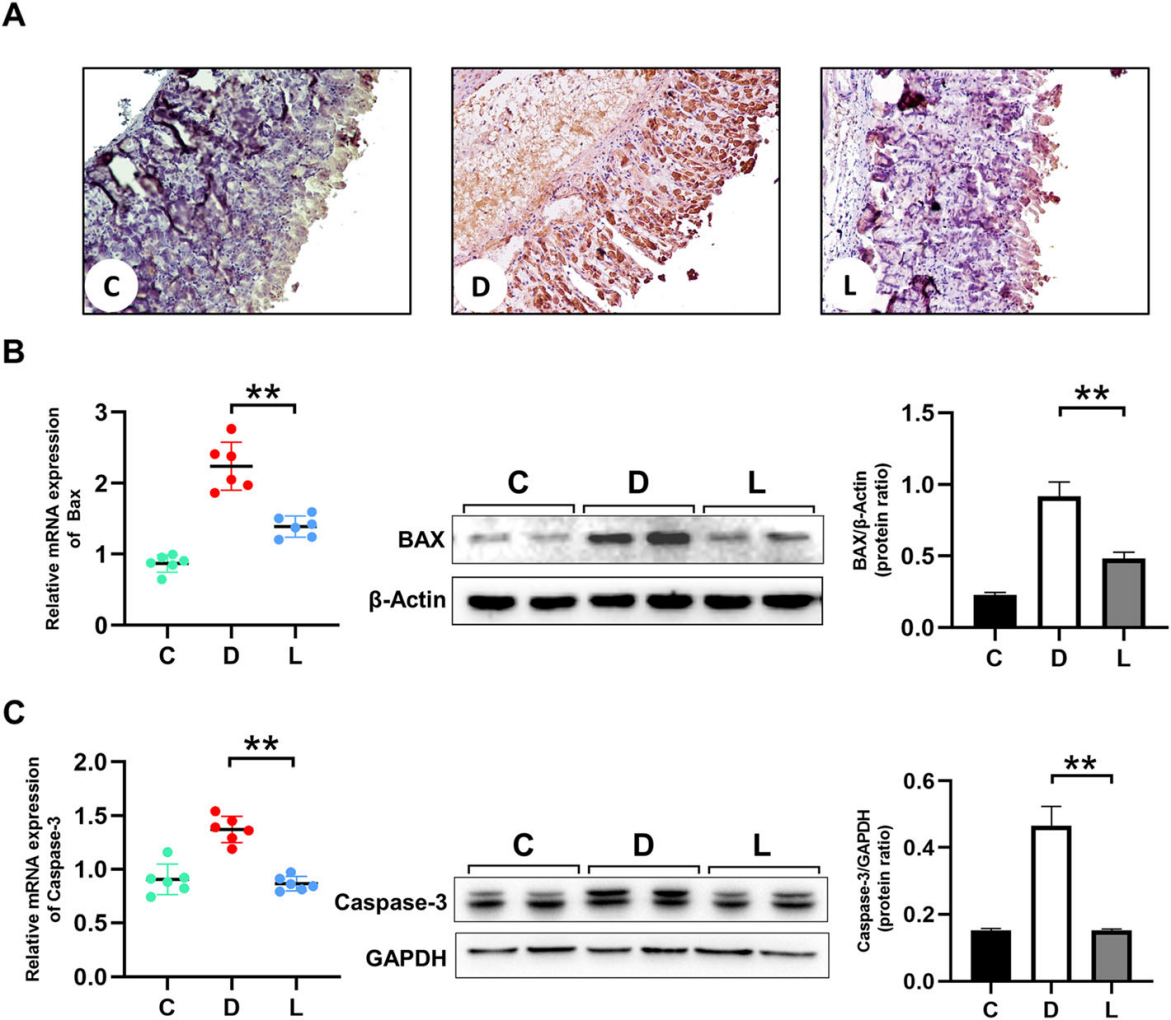
Effects of lactate on the apoptosis of gastric mucosa in ethanol-challenged mice. **a** IHC staining for Bax (× 20, scale bar = 100 μm). Gastric samples were fixed and sectioned for staining by the primary antibody of Bax. Brown granules in cells were considered positive results. **b** and **c** qPCR and western blot detection for Bax and Caspase 3, respectively. C: Control group; D: Disease group; L: animal group treated with lactate at 3 g/ kg. The data were expressed as the means ± SD (*n =* 6). ** *p* < 0.01

Immunohistochemical (IHC) analysis suggested an increased level of the apoptosis regulator Bax in the gastric mucosa of mice pre-administered with ethanol, compared with that of ethanol-free control animals. Pretreating animals with lactate counteracted such changes (Fig. 4a). In line with IHC results, western blot indicated that pretreatment of mice with lactate before the induction of GMI was associated with less production of Bax and Caspase 3, an apoptotic executive protein (Fig. 4b&c). qPCR confirmed above findings, showing down-regulation of the expression of *Bax* and *Casp3* that were induced by ethanol exposure (Fig. 4b &c).

### Lactate stimulated the expression of Occludin, Claudin-1, and Claudin-5 in the gastric mucosa of ethanol-challenged mice

**Fig. 5:**
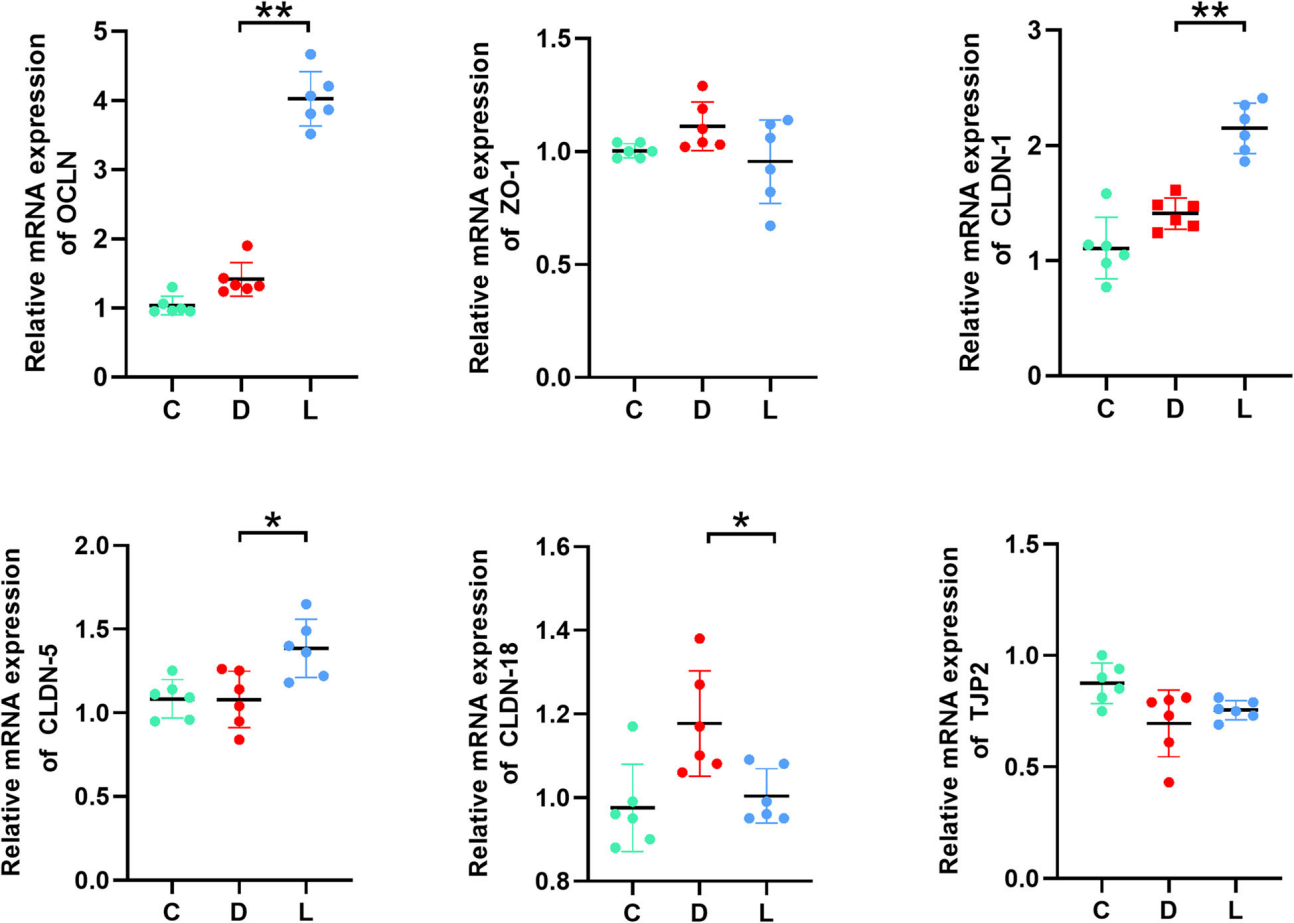
Effect of lactate on tight junction proteins of gastric tissues in ethanol-challenged mice. Total RNA in gastric tissues was extracted and qPCR was carried out to examine the expression of genes encoding ZO-1, Occludin, TJP2 and Claudin-1, − 5, − 18. C: Control group; D: disease group; L: Group treated with lactate at a dose of 3 g/ kg. The data were expressed as the means ± SD (*n =* 6). ** *p* < 0.01

*The tight junction proteins*, including Occludin, Claudins, and Zonula occludens, are crucial for the maintenance of gastric epithelial barrier *integrity*. qPCR was carried out to determine whether lactate modulated *tight junction integrity.* Lactate stimulated the expression of genes encoding tight junction proteins in the gastric tissues, including Occludin, Claudin-1, Claudin-5, but not Claudin-18, ZO-1 or TJP2 (Fig. 5).

### Lactate up-regulated GPR81 expression

**Fig. 6:**
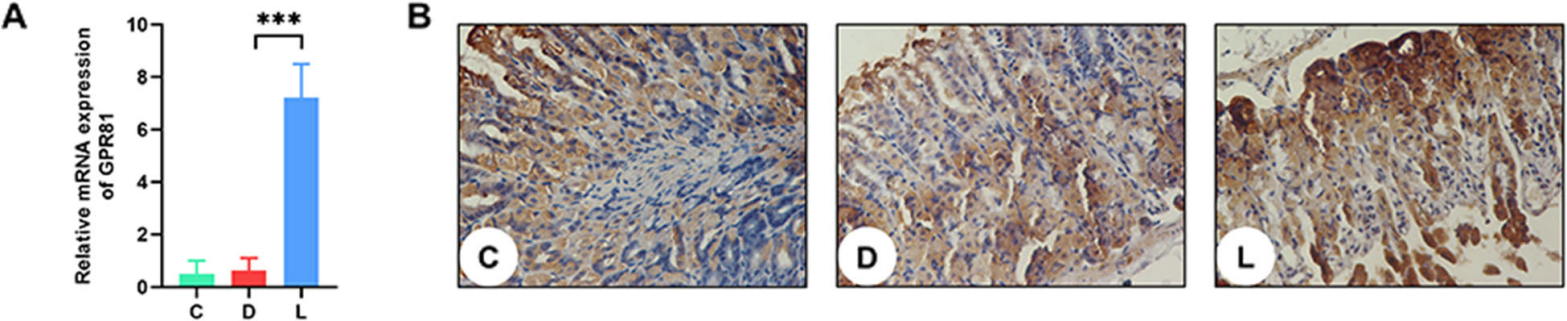
Expression of lactate receptor GPR81 in the various groups (*n =* 4 per group). **a** Total RNA in gastric tissues was extracted and qPCR was carried out to examine the expression of genes encoding GPR81. C: Control group; D: disease group; L: Group treated with lactate at a dose of 3 g/ kg. The data were expressed as the means ± SD (*n =* 6). ** *p* < 0.01. **b** IHC staining for GPR81. Gastric samples were fixed and sectioned for staining by the primary antibody of GPR81. Brown granules in cells were considered as positive results

Given that lactate receptors GPR81 is important for the biological function of lactate, we tested its expression upon exposure to lactate. qPCR results showed that the expression of GPR81 was significantly up-regulated by lactate treatment (*P* < 0.01; Fig. 6a). Consistently, IHC analysis showed more strongly expressed GPR81 in the lactate group as compared to the non-lactate control group (Fig. 6b).

**Fig. 7:**
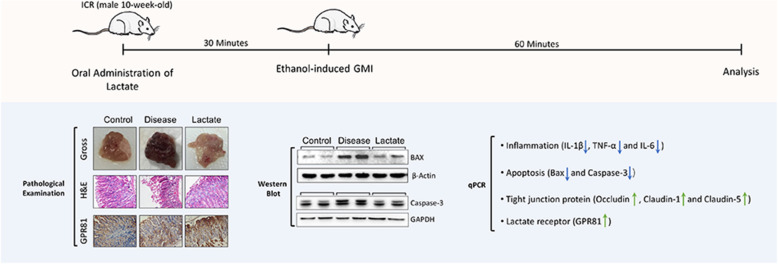
Schematic diagram of the protective mechanism. Lactate pretreatment attenuated the ethanol-induced GMI by inhibiting inflammation and apoptosis, and up-regulating tight junction proteins and the lactate receptor GPR81

## Discussion

Gastric mucosal lesions is a severe precancerous condition of gastric cancer which is one of the leading adenocarcinomas in many East Asian countries [3, 5, 26]. Probiotics have been recommended as an effective supplement to the current therapy of GMI using antisecretory drugs and antacid [2]. How probiotics exert their protective effects on GMI remains to be further uncovered. This study used an established mouse model to assess the effects of lactate, the major metabolite of *lactobacilli*, on gastric mucosa against ethanol-induced GMI. Key findings of this study include 1) lactate pre-administration significantly reduced the severity of ethanol-induced GMI in vivo; 2) lactate curtailed uncontrolled local inflammation encountered in GMI; 3) lactate interfered with the apoptosis in the gastric mucosa by down-regulating the expression of genes encoding apoptosis regulators Bax and Caspase 3; 4) lactate also up-regulated the expression of genes encoding defensive tight junction proteins in the gastric tissues, including Occludin, Claudin-1, and Claudin-5; 5) lactate up-regulated the expression of receptor GPR81.

Rodent models of ethanol-induced GMI are preferred in vivo assays for evaluating gastroprotective efficacy of potential compounds and to further study their underlying mechanisms [5, 27]. This model replicates the typical clinical manifestations of human GMI, such as bleeding and erosion of gastric mucosa [23, 24]. Numerous studies have reported that probiotic *Lactobacilli* were able to protect the gastric mucosa from ethanol-induced damage [12, 13, 28, 29]. Lactate is the major metabolite of probiotic *Lactobacilli*. Kahlert et al. (2016) used intestinal epithelial cell lines and reported in vitro gastrointestinal protective effect of lactate [30]. with the current study provided solid experimental evidence to support the in vivo gastroprotection of lactate against GMI. It should be noted that apart from *lactobacilli*, many other microbial species such as *Carnobacterium*, *Enterococcus*, *Tetragenococcus* may also produce lactate. Future studies should be carried out to examine any potential protective effect of these microorganisms on gastric mucosal injury. The gastroprotective effect of lactate against ethanol-induced GMI is possibly multifaceted, including its anti-inflammatory activity, anti-apoptosis potential, and its contribution to the integrity of gastric mucosal barrier. Our study found lactate was able to attenuate local inflammation in GMI, supported by the significantly lower level of IL-1β, TNF-α and IL-6 in gastric tissue samples. These pro-inflammatory factors have been widely used by others for evaluation of severity of GMI [5, 31]. Anti-inflammatory properties of lactate have been reported by Iraporda et al. (2016) and Ratter et al. (2018), using an in vivo trinitrobenzene sulfonic acid (TNBS)-induced colitis model and in vitro human primary peripheral blood mononuclear cells and monocyte cell cultures respectively [32, 33]. The underlying mechanisms, however, remain to be fully elucidated. It has been reported that lactate was able to activate its receptor GRP81 to inhibit inflammation in mice with colitis [20] or activate hydroxycarboxylic acid 2 (HCA2) to increase the survival of septic mice [34]. Our data showed that the expression of GPR81 in the stomach was significantly up-regulated by lactate treatment, suggesting an important role of GPR81 in the gastric protective effect of lactate. Apoptosis is another key player in the pathogenesis of ethanol-induced GMI [7]. Our study suggested that the anti-apoptotic effect of lactate might be related to the down-regulation of *Bax* expression and reduction in the activity of Caspase 3. Suppression of apoptosis by lactate may also attribute to its anti-inflammatory effects as apoptosis is known to be mediated by excessive inflammation [7, 8]. Gastric mucosal barrier is the first-line host defense against gastric mucosal damages [31, 34]. Studies by others have found a positive regulatory role of probiotic *Lactobacillus casei* or lactate on intestinal mucosal integrity, possibly by accelerating intestinal-stem-cell-mediated epithelial development [35, 36]. Tight junction proteins are key factors contributing to the integrity of epithelial layers of the gastric mucus [31, 37, 38]. Using tight junction proteins as a proxy for gastric mucosal integrity, we found lactate could promote the expression of OCLN and CLDN-1, CLDN-5, but not that of TJP2, ZO-1 or CLDN-18.

Probiotics have been trialed as a prophylactic or therapeutic supplement for human GMI, based on many in vivo studies that support their roles in tuning the complex gastric microbiota in the human stomach [29, 31, 34]. The current study, along with our previous work [5, 7] that examined the protective effects of short-chain fatty acids butyric acid and acetic acid, two metabolites of probiotics, on gastric mucosa, further rationalize the application of probiotics in combatting GMI.

## Conclusion

In summary, lactate, the main metabolite of *Lactobacilli* and some other microorganisms, has a protective effect on gastric mucosa against ethanol-induced injury, through anti-inflammation, anti-apoptosis, promoting the expression of tight junction proteins OCLN, CLDN-1, and CLDN-5 and up-regulating the expression of a lactate receptor GPR81. Our theoretical hypothesis regarding the protective mechanism of lactate is presented in Fig. 7. The findings of this study underpin the application of probiotics as a preventative or treatment strategy for GMI.

## Supplementary Information

**Additional file 1.**

**Additional file 2.**

**Additional file 3.**

## Data Availability

The datasets used and/or analyzed during the current study available from the corresponding author on reasonable request.

## Abbreviations

Bax: Bcl-2-associated X
BCA: Bicinchoninic acid
*Casp*: Caspase
CLDN: Claudin
DAB: Diaminobenzidine
ELISA: Enzyme-linked immunosorbent assay
EIA: Enzyme immunoassay
GAPDH: Glyceraldehyde-phosphate dehydrogenase
GMI: Gastric mucosal injury
HCA: Hydroxycarboxylic acid
HE: Hematoxylin–Eosin
HRP: Horseradish peroxidase
ICR: Institute of Cancer Research
IHC: Immunohistochemical
IL: Interleukin
OCLN: Occludin
PBST: Phosphate-buffered saline with tween
PVDF: Polyvinylidene fluoride
qPCR: Quantitative PCR
SCFA: Short-chain fatty acid
SDS-PAGE: Sodium dodecyl sulfate polyacrylamide gel electrophoresis
TNBS: Trinitrobenzene sulfonic acid
TNF: Tumor necrosis factor
TJP: Tight junction protein
