# Supplementary figures and images for: Lactate as a metabolite from probiotic Lactobacilli mitigates ethanol-induced gastric mucosal injury: an in vivo study
Source: BMC Complement Med Ther. 2021 Jan 11;21:26. doi: 10.1186/s12906-020-03198-7 (PMC7802211; doi:10.1186/s12906-020-03198-7)

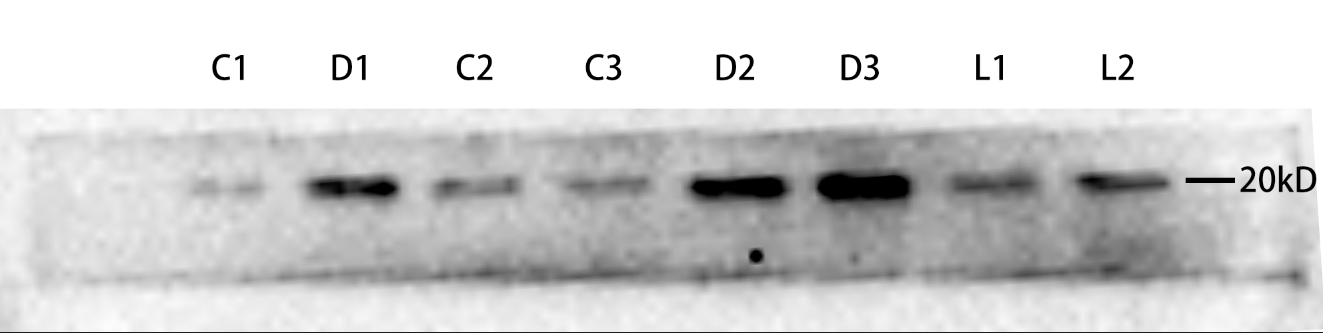

Supplement: Supplementary file 1 — Additional file 1. [file 12906_2020_3198_MOESM1_ESM.tif]

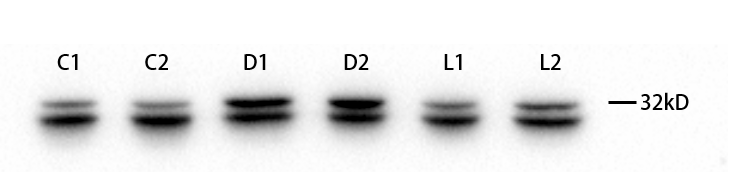

Supplement: Supplementary file 2 — Additional file 2. [file 12906_2020_3198_MOESM2_ESM.tif]

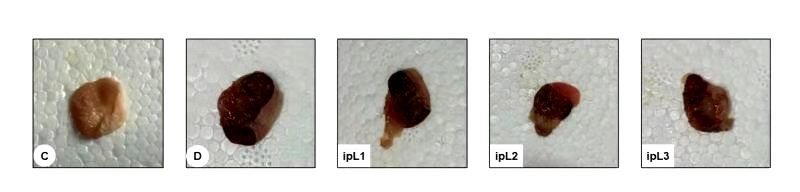

Supplement: Supplementary file 3 — Additional file 3. [file 12906_2020_3198_MOESM3_ESM.tif]
